# Evaluation of a multimodal pain therapy concept for chronic pain after total knee arthroplasty: a pilot study in 21 patients

**DOI:** 10.1186/s13037-017-0137-x

**Published:** 2017-08-30

**Authors:** Dirk Zajonz, Johannes K. M. Fakler, Anna-Judith Dahse, Fujiaoshou Junping Zhao, Melanie Edel, Christoph Josten, Andreas Roth

**Affiliations:** 1 Department of Orthopaedic Surgery, Traumatology and Plastic Surgery, University Hospital Leipzig, Liebigstrasse 20, D-04103 Leipzig, Germany; 2 Pharmacy of the University Hospital Leipzig, Liebigstrasse 20, D-04103 Leipzig, Germany; 3 Clinic for Anesthesiology and Intensive Therapy, University Hospital Leipzig, Liebigstrasse 20, D-04103 Leipzig, Germany; 4 ZESBO – Zentrum zur Erforschung der Stuetz- und BewegungsOrgane, University of Leipzig, Semmelweisstrasse 14, D-04103 Leipzig, Germany

**Keywords:** Arthrofibrosis, Multimodal pain therapy, Total knee arthroplasty, Ketamine, Traditional Chinese medicine

## Abstract

**Background:**

In spite of the improvement of many aspects around Total knee arthroplasty (TKA), there is still a group of 10% to 34% of patients who is not satisfied with the outcome. The therapy of chronic pain after TKA remains a medical challenge that requires an interdisciplinary therapy concept. The aim of this prospective pilot study was to evaluate the efficacy of a multimodal pain therapy in chronic complaints after TKA.

**Methods:**

In a prospective cohort pilot study, we included patients with chronic pain after TKA who obtained in-patient care, especially multimodal pain therapy (MMPT), for at least 10 days. Essential elements of this therapy concept were physiotherapy, pain medication therapy, topical application of ketamine, local infiltration and Traditional Chinese Medicine. Patients with varying causes of complaints were excluded in advance. Before the start of the study all test persons were informed and gave their written consent. Moreover, each patient was examined and questioned at hospital admission, discharge and at its first as well as second follow-up. Additionally, knee joint mobility and stability were investigated at all examination times.

**Results:**

From 03/07/2016 to 07/14/2016, 21 patients were included in the pilot study. 52% of the considered population were female (11 persons). The median age was 65 years (45–79 years) and the median stay in hospital amounted 9 days (8–14 days). The first follow-up was scheduled after six weeks (median: 38 days, 30–112 days) and the second one after six months (median: 8 months, 7–12 months). The number of patients of the first follow-up was 17 out of 21 (19% drop out). The drop out of the last follow-up accounted for 33%. All patients benefit from the presented applications and therapies with regard to pain, function and range of motion. Especially, during the period of in-patient treatment, nearly all patients have improved in all terms. However, during the first follow-up clear deteriorations occurred in all areas, which stagnated up to the second follow-up. The range of motion has even worsened slightly.

**Conclusions:**

With the presented pilot study on multimodal in-patient therapy of chronic complaints due to TKA, the improvement of pain, function and mobility could be verified, especially during the stationary stay. Even though the results deteriorate during the follow-up period, they did never relapse to their initial level. In order to ensure an effective treatment, a clear diagnostic algorithm is essential, by which treatable causes, such as low-grade infections or loosenings, are safely excluded. Further prospective studies are necessary to obtain precise statements on prospects of success of our therapy plan.

## Background

Osteoarthritis of the knee is a common condition that affects approximately 10% of the general population above the age of 60 years [1, 2]. Frequently, the diagnosis is based on radiological findings. In a study of knee osteoarthritis by Kellgren and Lawrence from 1958 a prevalence of 40.7% in females and 29.8% in males aged 55 to 65 years was found [3]. However, correlations between radiological findings and clinical symptoms are only possible to a limited extent. Spector et al. determined symptomatic radiographic knee osteoarthrosis in 2.9% of women of this age range [4]. With increasing age the prevalence of symptomatic knee osteoarthrosis rises up to 40% in the group aged from 75 to 79 years [5]. Conservative therapy, like non-steroidal anti-inflammatory drug administration, joint injections, stabilizing physiotherapy and weight reduction, is the most common treatment of symptomatic knee osteoarthrosis [6]. As a result, the progress of the disease can be slowed down and symptoms can be alleviated. When conservative therapy has been exhausted, partial or complete artificial joint replacement has established as a gold standard [7]. Total knee arthroplasty (TKA) is an effective way to manage end-stage knee osteoarthritis, to relieve pain and achieve functional improvements [8–10]. Over the years, many refinements have been made to this surgical procedure in order to improve patient outcomes, reduce postoperative complications and ultimately to enhance the patient’s life quality after TKA [2, 11]. In spite of the improvement of many aspects around TKA, there is still a group of patients who is not satisfied with the outcome. In an extensive meta-analysis of 1308 articles, 115 patient-centered pain outcomes were reported, whereof the proportion of people with an unfavorable long-term pain outcome after TKA ranged from 10% to 34% (median: 20%) [10]. The causes of these persistent complaints are manifold and often multifactorial. There are rarely clear causes, such as mechanical problems, low-grade infections, premature loosening or cement and metal hypersensitivities, respectively [12–16]. Extensive diagnostics are also not always able to find a definite cause. Finally, many factors remain unclear. This often results in frequent conservative and operative therapies without significant improvements up to a total knee arthroplasty replacement (TKAR). Consequently, the therapy of chronic pain after TKA represents a medical challenge that requires an interdisciplinary therapy concept. The concept of multimodal pain therapy (MMPT), especially for the treatment of chronic lower back pain, is already clinically proven and well evaluated by studies [17, 18]. Therefore, it is obvious to transfer this concept to patients with chronic complaints after TKA.

The aim of this prospective pilot study was to evaluate the efficacy of a multimodal pain therapy of chronic complaints after TKA. Depending on the results of this pilot study, a prospective two-armed survey is planned for further evaluation.

## Methods

In a prospective cohort pilot study 21 patients with chronic pain after TKA were treated stationary by multimodal pain therapy (MMPT) for at least 10 days. All patients with differing causes of complaints were excluded in advance. In order to safely preclude implant loosening, inlay wear or other existing mechanical complications radiographs were used. A joint infection was excluded by blood tests and knee joint puncture. In individual cases further investigations (allergy tests, nuclear medical examinations, sectional imaging, etc.) were carried out. In order to increase the accuracy of an exclusion of periprosthetic infections, a previous arthroscopy with 5 tissue samples is planned. This can also be used to confirm a suspicious diagnosis like an arthrofibrosis by means of tissue analysis.

During the planning of the study, it was discussed with the local ethics committee. Since the investigation has been assessed as an individual test of salvation, there is no provision for the ethics committee. An ethics vote is planned for the randomized follow-up study. Before the beginning of the study all patients were informed and gave their written consent to the off-label use of the medication as an individual therapeutic attempt as well as to the publication of data.

For medication, the subjects received 20 mg of prednisolone (Reduction of inflammation and pain modulatory effects), 10 mg of propranolol (influences of sympathetic tone and reflexive pain modulation) and 25 mg of amitriptyline (modulation of neuralgiform pain) once a day over a period of 25 days. After initial exclusion of contraindications for this medication daily blood glucose control as well as measurements of pulse and blood pressure were performed. In addition, patients were treated with topical ketamine (10% ketamine hydrochloride in pre-mixed PLO gel 30, MEDISCA, USA).) Ketamine hydrochloride was used as a pure substance according to the European Pharmacopoeia (Ph.Eur.). PLO gel 30 is a ready-to-use transdermal base, composed of an oily phase (lecithin and isopropyl palmitate) and an aqueous phase (Pluronic gel 30%). The advantage of this organogel is that high amounts of active ingredients can be incorporated by the preparation which can easily pass through the skin into deeper tissues.

**Fig. 1:**
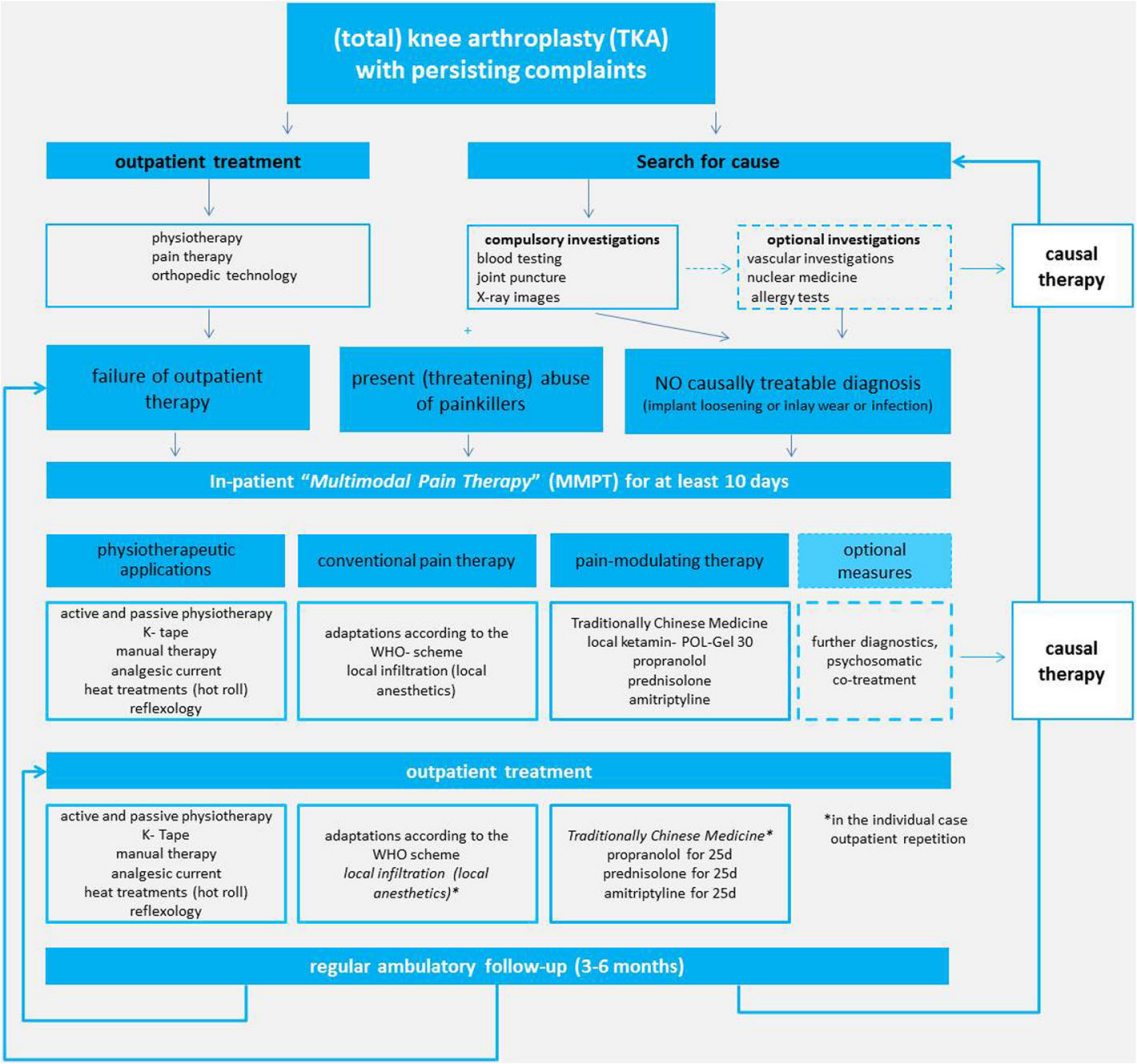
Scheme showing the treatment algorithm of the University Hospital Leipzig for persistent complaints after total knee arthroplasty

In the course of in-patient therapy, all patients also received regular treatments in Traditional Chinese Medicine (TCM) with acupuncture, acupressure and cupping. Moreover, daily physiotherapeutic applications with passive physiotherapy, thermal applications (hot roller), reflexology, manual therapy and Kinesiotape were performed. The pain relief service was used to optimize pain therapy. Local infiltration of points of pain with local anesthetics (lidocaine 1% and bupivacaine 0,5%) was applied, too. Figure 1 describes the treatment algorithm (kind of decision tree) of the University Hospital Leipzig relating to the therapy of persistent complaints after TKA.

All patients were examined and questioned at the time of hospital admission, discharge and at their first as well as second follow-up. Knee joint mobility and stability were investigated at all examination times. In this context, the following scores were applied.

- Numerical Analog Scale (NAS) for pain ranging from 0 to 10 with 0: no pain and 10: worst imaginable pain.
- Knee Score of Ranawat and Shine (Hospital for Special Surgery Score, HSS Score) ranging from 0 to 100 with <60: inadequate, 60–69: moderate, 70–84: good and 85–100: excellent. The score includes pain 30%, function 22%, range of motion 18%, muscular strength 10%, flexion deficit 10% and stability 10% [19].
- Knee Society Score of Insall et al. consisting of a clinical (knee) score (50%) and a function score (50%), both ranging from 0 to 100. The score includes pain 25%, range of motion 12.5%, stability 12.5%, walking distance 25% and stairs 25% [19].

## Results

From 03/07/2016 to 07/14/2016, 21 patients were included in the pilot study. 11 of them were women (52%). The median age was 65 years (45–79 years). With regard to the median, primary TKA implantation took place 5 years ago (1–12 years). The median value of patient’s previous operations amounted 2 treatments (1–6 operations) including primary TKA. 16 patients (76%) had already received at least one change of TKA (1–3 changes). Concerning the median, the last operation has been performed 12 months (4–84 months) ago.

Moreover, the median stay in hospital was 9 days (8–14 days). The first follow-up was planned after six weeks (median: 38 days, 30–112 days) and the second one after six months (median: 8 months, 7–12 months). The first follow-up included 17 out of 21 patients (19% drop out), whereas the following one showed a drop out of 33% (14/21).

**Fig. 2:**
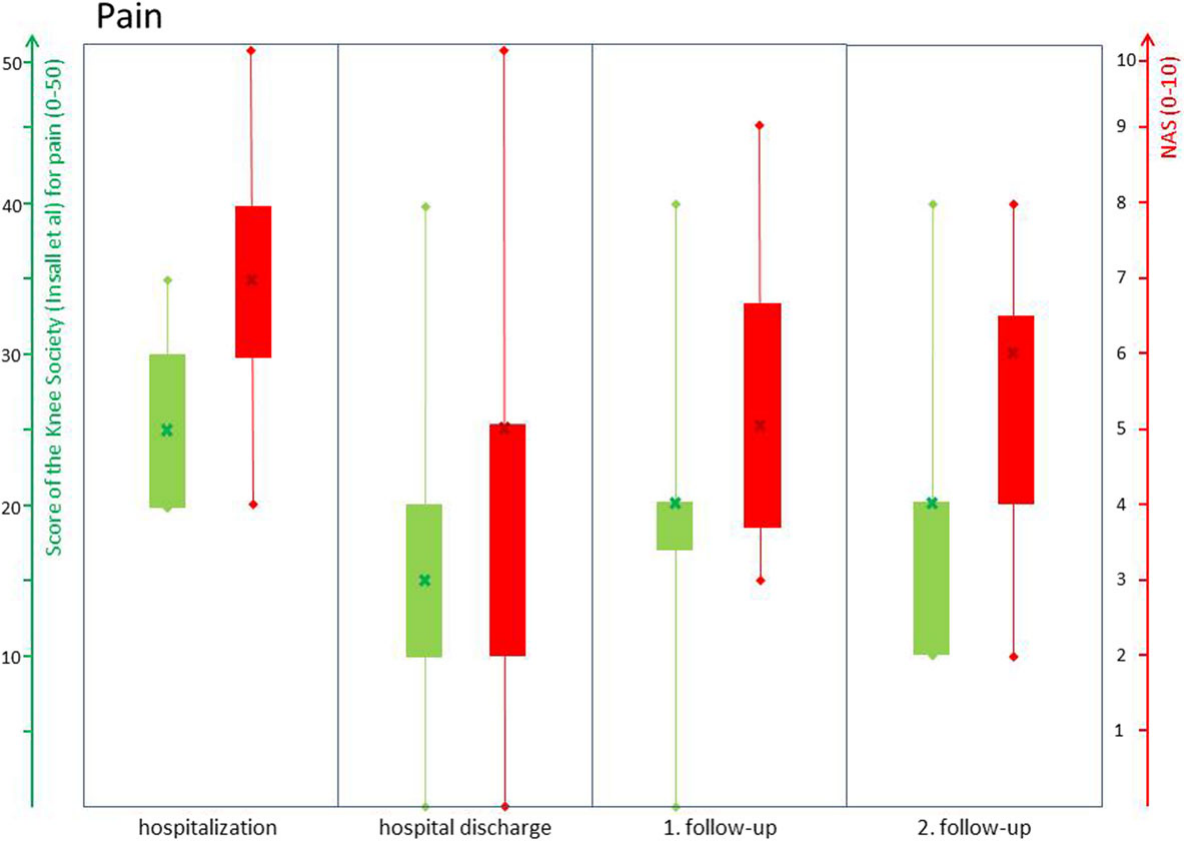
Pain development at the times of examination (hospitalization, hospital discharge, 1. follow-up, 2. follow-up). *Green*: Knee Society Score of Insall et al. for pain (0–50); *Red*: Numerical Analog Scale for pain (0–10)

**Fig. 3:**
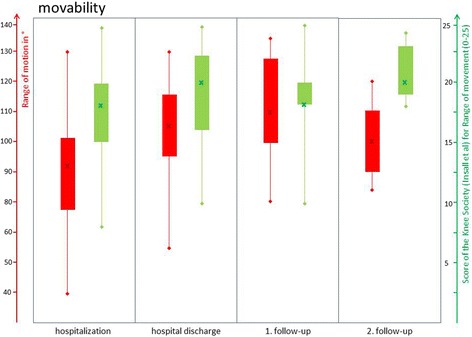
Visualization of change in range of motion at the times of examination (hospitalization, hospital discharge, 1. follow-up, 2. follow-up). *Red*: neutral zero method in degree; *Green*: Knee Society Score of Insall et al. for range of motion (0–25)

**Fig. 4:**
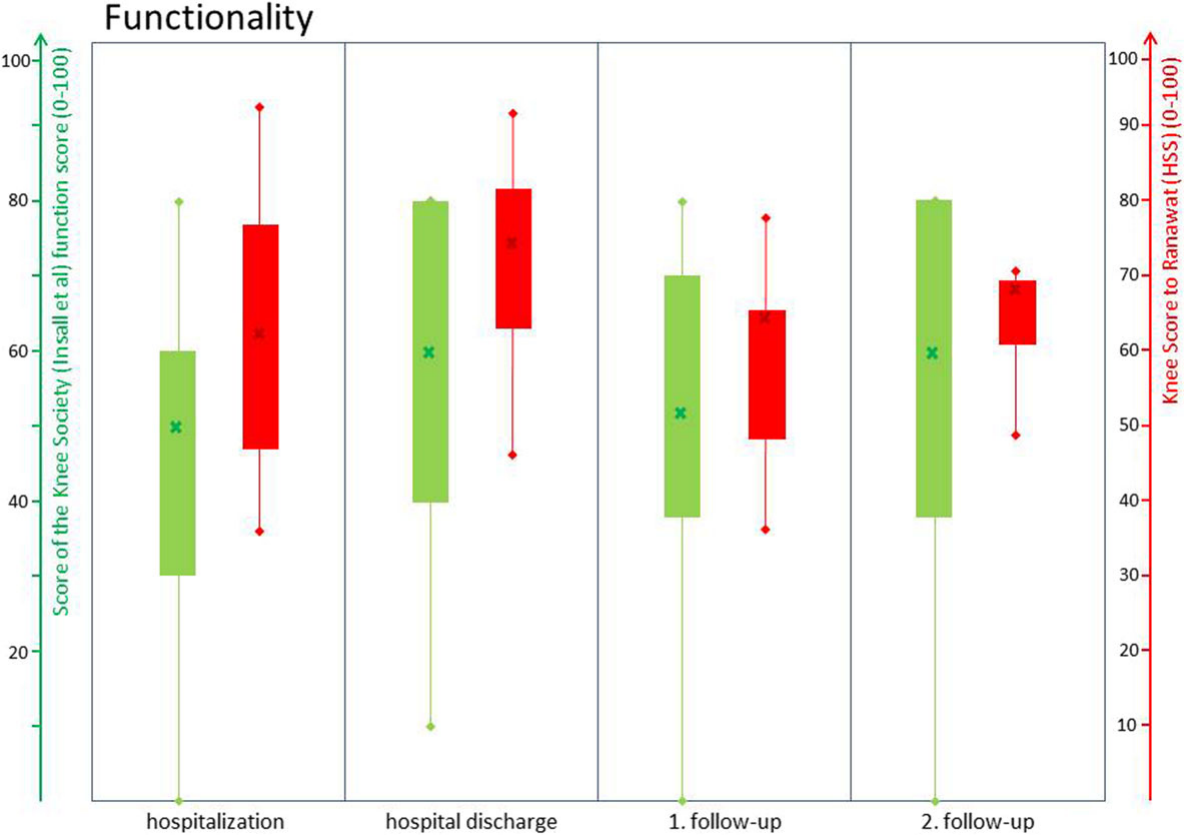
Graphical description of functional changes at the times of examination (hospitalization, hospital discharge, 1. follow-up, 2. follow-up). *Green*: Function Score of the Knee Society Score of Insall et al. (0–100); *Red*: Knee Score of Ranawat and Shine for functionality (0–100)

In this context, Table 1 provides an appropriate overview of patient’s specifics according to sex, age and relevant medical history. Table 2 summarizes the median values at all examinations times of each evaluation implemented by the Numerical Analog Scale, the Knee Society Score of Insall et al., the Knee Score of Ranawat and Shine and the neutral zero method (ROM), respectively. In the median, the patients reported an improvement on the NAS (7 to 5) and the Knee Society Score of Insall (pain 25 to 15) in terms of pain during hospitalization. Both markers have increased to the 2nd follow up, but have not reached the initial level again. All changes were without statistical significance. Figure 2 illustrates the development of the pain history at all examination times Fig. 3 shows the change in movability measured by the neutral zero method (ROM) and the Knee Society Score of Insall et al. Here too, the patients in the median showed an improvement in the range of motion, especially during stationary observation (92 to 105). The ROM increased to the first follow up, but then fell again for the second follow-up. All changes were without statistical significance. The same trend is also shown with regard to the functionality where the drop down to the 2 follow up was not so impressive here and the patients were stable to the level. Patient’s functional changes assessed by the relevant subscore of Insall et al. (function score) as well as the Knee Score of Ranawat and Shine are represented graphically by Fig. 4. All changes were without statistical significance.

**Table 1:** Presentation of the specifics of the patient collective with sex (0: male, 1: female), age, year of primary implantation, number of previous operations, number of TKA changes and time elapsed since last operation

Patient number	Female	Age (years)	Year of primary implantation	Number of preoperations / revisions	Number of TKA changes	Time to last surgery (months)
1	1	60	2004	5	2	23
2	0	79	2013	5	1	12
3	1	65	2008	5	2	30
4	0	59	2012	3	1	15
5	1	57	2008	2	1	84
6	1	60	2011	2	0	7
7	1	56	2014	not specified	2	5
8	0	45	2009	3	2	11
9	1	71	2007	1	not specified	24
10	0	68	2012	4	2	8
11	1	74	2011	3	2	24
12	0	78	2010	2	1	36
13	0	67	2014	not specified	2	4
14	1	68	2015	2	0	13
15	1	57	2009	1	0	24
16	1	47	2008	2	0	4
17	1	62	2009	2	1	14
18	0	71	2012	2	1	11
19	0	77	2009	3	1	9
20	0	70	2015	2	1	4
21	0	52	2011	6	3	not specified

**Table 2:** Summary of score-specific median values including their ranges at the times of examination (hospitalization, hospital discharge, 1. follow-up, 2. follow-up). Numerical Analog Scale for pain (0–10); Knee Society Score of Insall et al. (0–200), which is subdivided into a clinical score (pain, ROM, stability) and a function score (walking, stairs); Knee Score of Ranawat and Shine (0–100), which is divided into <60: inadequate, 60–69: moderate, 70–84: good and 85–100: excellent; neutral zero method for ROM indicated in degrees

	NAS	Knee Society Score of Insall	Pain	Movment	Stability	Clinical Score	Walking	Stairs	Function score	Knee Score of Ranawat (HSS)	Excellent	Good	Moderate	Inadequate	Range of motion
Hospitalisation	7 (4–10)	88 (14–145)	25 (20–45)	18 (8–25)	25 (20–25)	45 (13–85)	20 (10–50)	30 (0–30)	50 (10–80)	62 (36–95)	5%	33%	19%	43%	92,5 (40–130)
Hos. discharge	5 (0–10)	118 (55–160)	15 (0–40)	20 (10–25)	25 (10–25)	59 (25–80)	30 (10–50)	30 (15–40)	60 (15–80)	74 (46–92)	23.50%	41.50%	17.50%	17.50%	105 (55–130)
1st follow-up	5 (3–9)	112 (55–168)	20 (0–40)	18 (10–25)	25 (20–25)	62 (25–88)	25 (10–40)	30 (0–40)	52,5 (0–80)	64 (36–77)	0	20%	40%	40%	110 (80–135)
2nd follow-up	6 (2–8)	119 (43–168)	20 (10–40)	20 (18–24)	25 (20–25)	63 (43–88)	30 (10–40)	40 (0–40)	60 (0–80)	67 (49–70)	0	30%	40%	30%	100 (85–120)

## Discussion

The treatment of patients with chronic complaints after TKA is a challenging task. Therefore, an adequate therapy is only possible due to an interdisciplinary team of experienced orthopaedic surgeons with great knowledge in the field of endoprosthetics, qualified physiotherapists and pain therapists.

### Traditional Chinese medicine (TCM)

As a part of the MMPT the application of TCM had positive influence on the therapeutic process. Many patients described these treatments as particularly effective. Yang et al. proved a significant alleviation of pain and diminution of flexion contractures achieved by TCM [20]. Specifically, the use of acupuncture and acupressure were indicated as long-term pain relief on the part of patients. The efficacy of acupuncture during post-acute phase of rehabilitation after total knee arthroplasty with respect to pain relief has already been reported by Mikashima et al. and Chen et al. They also showed that TCM leads to a reduction of knee swelling and an early recovery of ROM [21, 22]. However, studies on success rates of TCM treating chronic complaints after TKA are not available.

### Additional pain medication

In most patients, a chronic abuse of pain medication was present. Therefore, we have optimized and adapted this medication application according to the WHO model with the assistance of the internal pain service. The above-described supplementary drug therapy was applied in accordance with the treatment success of Dr. Philipp Traut [23, 24]. He was able to establish himself in Germany, especially in the area of conservative therapy of patients with arthrofibrosis due to TKA. The effect of amitriptyline in neuropathic pain is well documented and established in clinical use [25]. The use of oral prednisolone is described in the treatment of back pain and multimodal pain therapy concepts [26, 27]. Prednisolone reduces the chronic inflammatory reaction and an increased expression of cytokines in arthrofibrotic tissue [28]. The dosage and duration went back to the works of Traut [29]. Traut carries out prednisolontherapy from 20 to 30 days completely stationary. He also reports that patients with narcotic mobilizations or scar excision in the prehistory and extreme fibrosis sometimes require repetition of prednisolone medication [30]. Propranolol in pain therapy is most commonly described in migraine [31]. Some clinical studies have shown opioid-sparing effects of β-blockers are being investigated for chronic musculoskeletal pain. Schweinhardt showed decreased pain sensitivity by Propranolol with analgesic effects [32]. Moreover, it is important that the medication therapy with prednisolone and propranolol takes all contraindications as well as side effects into account. Consequently, regular blood glucose and blood pressure checks must be performed. Apart from all these factors, the duration of therapy may not be exceeded and the medication shouldn’t discontinued abruptly, too. A combination with other beta blockers of patient’s home medication should also be avoided. When using prednisolone, an infection should be excluded first (knee joint puncture and x-ray thorax, blood samples). Nevertheless, it should be mentioned that there are no studies on the success of the described drug therapy. For that reason, all patients should be completely informed about the medication’s off-label use.

### Topical ketamine

Peripheral nociceptors express n-methyl-d-aspartate (NMDA) receptors (NR1, NR2B) which are involved in the modulation of pain delivery. However, their systemic use is limited by adverse effects [33]. Nevertheless, the use of topical ketamine has been described as a tried and tested remedy. A significant reduction of mechanical hyperalgesia was generated by topically and pre-emptively applied ketamine in healthy patients [33, 34]. However, in order to avoid transdermal penetration of active ingredients gloves have to be worn during the application or physical contact in general.

### Physiotherapy

The broad field of physiological gymnastics represents an important part of the MMPT. Particularly in the therapy of arthrofibrosis it is known that forced exercises can aggravate adhesions [35]. Therefore, we have refrained from intensive exercises to improve the movement in favor of pain-relief applications. Above all, passive applications, such as lymph drainage, reflexology, Kinesiotape and trigger points, have proven to be effective treatments [36–39]. In the course of these passive procedures patients’ mobility has also improved, especially during their hospitalization without active or passive treatment of mobility. In this context, it is important that physiotherapy will be continued after discharge in an out-patient setting.

### Local infiltration analgesia

Particularly in immediate postoperative pain therapy after TKA, local infiltration analgesia (lidocaine 1% and bupivacaine 0,5%) has significantly reduced the length of stay and the postoperative pain scores [40]. But, it has been reported that it has an analgesic effect of less than 24 h [41]. Therefore, it should be primarily used for short-term pain therapy. Taken this into account, local infiltration analgesia should be applied, for example, shortly before the beginning of physiotherapeutic treatments or as diagnostic injection. In individual cases, a repetition can be useful.

### Psychosomatic support

Ideally, the treatment should be supported by psychotherapists or psychologists with experiences in the area of psychosomatic medicine. Psychosomatic factors associated with individual pain relief and processing have already been extensively investigated [42]. In this context, it is known that the implementation of psychological treatment has become a well-established feature of the MMPT of patients with chronic lower back pain. [17, 43]. Ali et al. proved that preoperative anxiety and depressions are important predictors for dissatisfaction after TKA [44, 45]. It is verified that next to medical also psychological comorbidity predicts poor pain outcomes after total knee arthroplasty [46]. According to this, in two cases (suicidal thoughts due to chronic pain, suspicion of manipulation) we have consulted the colleagues of psychosomatics with good individual success. However, it was not possible to offer regular psychosomatic treatment during this pilot study. For this reason, further studies will take this into account.

All patients benefit from the presented applications and therapies with regard to pain (Fig. 2), function (Fig. 4) and range of motion (Fig. 3). Especially, during the period of in-patient treatment, nearly all patients have improved in all terms. (Figs. 2, 3 and 4) However, during the first follow-up (median: 38 days) clear deteriorations occurred in all areas, which stagnated up to the second follow-up (median: 8 months). The range of motion has even worsened slightly. (Figs. 2, 3 and 4) Nonetheless, the baseline conditions were improved after eight months in all subgroups. Therefore, it can be assumed that the follow-up patients have benefited from in-patient therapy. But these results do not show any statistical significance due to the small collective size of the pilot study. Furthermore, it must be expected that the results are positively distorted by the high drop-out rate (33% after eight months). The probability of developing an arthrofibrosis increases with the number of preoperations [47]. In this case, invasive operations (prosthesis replacement) have a higher potential than small procedures (arthroscopies) for the development of an arthrofibrosis. This is caused by the activation of massive connective tissue proliferation (collagen VI) which increases with each operation [48]. (The study size of this pilot study is too small to establish a correlation between the number or type of the preoperations on the extent of the arthrofibrosis. Regardless of the therapy, a clear diagnosis which enables a definite exclusion of treatable causes is essential. In our pilot study, two low-grade infections and two aseptic loosenings were shown, despite an initial inconspicuous diagnosis. For that reason, it is useful to follow a distinct diagnostic algorithm in terms of making a correct diagnosis according to painful TKA and recommending suitable therapies However, up to 10% to 15% of patients with residual pain may have unexplained pain [49]. Especially for these patients, further research on treatment programs for chronic TKA complaints seems indispensable.

## Conclusions

With the presented pilot study on multimodal in-patient therapy of chronic complaints according to TKA, the improvement of pain, function and mobility could be shown. While the results deteriorate after hospital discharge, they rebalanced during the follow-up period with no relapse to their initial level. Further prospective studies are necessary to obtain precise statements on the success prospects through the therapy plan of the University Hospital Leipzig.

## Abbreviations

HSS: Hospital for Special Surgery
MMPT: Multimodal pain therapy
NAS: Numerical Analog Scale
NMDA: N-methyl-d-aspartate
PLO: Pluronic lecithin organogel
ROM: Range of motion
TCM: Traditional Chinese Medicine
THA: Total hip arthroplasty
TKA: Total knee arthroplasty
TKAR: Total knee arthroplasty revision
